# Relevance of HCN2-expressing human mesenchymal stem cells for the generation of biological pacemakers

**DOI:** 10.1186/s13287-016-0326-z

**Published:** 2016-04-30

**Authors:** Ieva Bruzauskaite, Daiva Bironaite, Edvardas Bagdonas, Vytenis Arvydas Skeberdis, Jaroslav Denkovskij, Tomas Tamulevicius, Valentinas Uvarovas, Eiva Bernotiene

**Affiliations:** Department of Regenerative Medicine, State Research Institute Centre for Innovative Medicine, Vilnius, Lithuania; Department of Pathology, Forensic Medicine and Pharmacology, Vilnius University, Faculty of Medicine, Vilnius, Lithuania; Institute of Cardiology, Lithuanian University of Health Sciences, Kaunas, Lithuania; Institute of Materials Science, Kaunas University of Technology, Kaunas, Lithuania; Clinic of Rheumatology, Orthopedic and Traumatology and Reconstructive Surgery, Faculty of Medicine, Vilnius University, Vilnius, Lithuania

**Keywords:** HCN2, Stem cells, Scaffold, Tissue regeneration, Biological pacemaker

## Abstract

**Background:**

The transfection of human mesenchymal stem cells (hMSCs) with the hyperpolarization-activated cyclic nucleotide-gated ion channel 2 (HCN2) gene has been demonstrated to provide biological pacing in dogs with complete heart block. The mechanism appears to be the generation of the ion current (I_f_) by the HCN2-expressing hMSCs. However, it is not clear how the transfection process and/or the HCN2 gene affect the growth functions of the hMSCs. Therefore, we investigated survival, proliferation, cell cycle, and growth on a Kapton® scaffold of HCN2-expressing hMSCs.

**Methods:**

hMSCs were isolated from the bone marrow of healthy volunteers applying a selective cell adhesion procedure and were identified by their expression of specific surface markers. Cells from passages 2–3 were transfected by electroporation using commercial transfection kits and a pIRES2-EGFP vector carrying the pacemaker gene, mouse HCN2 (mHCN2). Transfection efficiency was confirmed by enhanced green fluorescent protein (EGFP) fluorescence, quantitative real-time polymerase chain reaction (RT-qPCR) and enzyme-linked immunosorbent assay (ELISA). After hMSCs were transfected, their viability, proliferation, I_f_ generation, apoptosis, cell cycle, and expression of transcription factors were measured and compared with non-transfected cells and cells transfected with pIRES2-EGFP vector alone.

**Results:**

Intracellular mHCN2 expression after transfection increased from 22.14 to 62.66 ng/mg protein (*p* < 0.05). Transfection efficiency was 45 ± 5 %. The viability of mHCN2-transfected cells was 82 ± 5 %; they grew stably for more than 3 weeks and induced I_f_ current. mHCN2-transfected cells had low mitotic activity (10.4 ± 1.24 % in G2/M and 83.6 ± 2.5 % in G1 phases) as compared with non-transfected cells (52–53 % in G2/M and 31–35 % in G1 phases). Transfected cells showed increased activation of nine cell cycle-regulating transcription factors: the most prominent upregulation was of AMP-dependent transcription factor ATF3 (7.11-fold, *p* = 0.00056) which regulates the G1 phase. mHCN2-expressing hMSCs were attached and made anchorage-dependent connection with other cells without transmigration through a 12.7-μm thick Kapton® HN film with micromachined 1–3 μm diameter pores.

**Conclusions:**

mHCN2-expressing hMSCs preserved the major cell functions required for the generation of biological pacemakers: high viability, functional activity, but low proliferation rate through the arrest of cell cycle in the G1 phase. mHCN2-expressing hMSCs attached and grew on a Kapton® scaffold without transmigration, confirming the relevance of these cells for the generation of biological pacemakers.

## Background

Electronic pacing is the state-of-the art treatment for complete heart block and a variety of other cardiac arrhythmias. While they successfully regulate heart rhythm, electronic pacemakers are not without risk, including infection, lead fracture and displacement, and potential interference from other devices. These concerns plus the need for frequent monitoring and for battery replacement have led to a search for other means—such as biological pacemakers—to provide cardiac pacing.

Some of these other means reported to provide biological pacing are virally delivered gene therapies aimed at increasing inward and or decreasing outward ionic currents during diastole, the use of transcription factors to convert mature myocytes into sinus node-like cells, and the use of embryonic stem cells (ESCs) or induced pluripotent stem cells (iPSCs) converted into a pacemaker lineage [1–5]. Another approach used mesenchymal stem cells (MSCs) and a self-inactivating HIV-based lentiviral vector for delivery of human potassium/sodium hyperpolarization-activated cyclic nucleotide-gated ion channel 2 (HCN2) into rabbit MSCs [6]. Bone marrow (BM)-derived human mesenchymal stem cells (hMSCs) transfected with the mouse HCN2 (mHCN2) gene were grafted to initiate a biological rhythm in the canine heart [7]. However, the MSCs have been shown to migrate away from the administration site within weeks of engraftment, making this a short-term solution. This problem might solved by trapping HCN2-expressing and heart rhythm-stimulating cells in cages/scaffolds thus preventing transmigration, preserving their long-term growth, sufficient nutrition and functional properties. Additionally, cell functions such as viability, stability, and renewal rate of HCN2-transfected cells are important factors.

Therefore, in the present study we explored the relevance of mHCN2-expressing hMSCs for the generation of biological pacemakers, paying particular attention to cell proliferation and cell cycle regulation and to the ability of mHCN2-transfected cells to grow and function on the porous Kapton® scaffold.

## Methods

### Isolation and expansion of BM-derived hMSCs

hMSCs were isolated, identified, and expanded from BM aspirates following previously described protocols [8, 9]. Briefly, BM aspirates (~6 ml) from the iliac crest of humans (up to 40 years old) were collected after obtaining written informed consent according to the Declaration of Helsinki and approval by the Lithuanian Bioethics Committee (No. 158200-14-741-257). BM donors were tested according to guidelines for the preparation of blood and blood products. Unprocessed bone marrow was seeded at a density of 12,000 mononuclear cells (MNC)/cm^2^ in T-75 flasks (NUNC, Wiesbaden, Germany) and cultured in Dulbecco’s modified Eagle’s medium (DMEM) containing 10 % fetal bovine serum (FBS) at 37 °C in a humidified atmosphere of 5 % CO_2_. After 24 h, supernatant containing non-adherent cells was removed, cells were rinsed with phosphate-buffered saline (PBS) without Ca^2+^/Mg^2+^ (Biochrom, UK) and growth medium was added. Additional changes of growth medium were performed every 3 days. For further experiments, cells were used from passages 2–3. Only the MSCs that corresponded to all main MSC identification criteria were included in the study.

### Transfection of hMSC with the mHCN2 gene

A full-length of mHCN2 cDNA, subcloned in a pIRES2-EGFP vector (BD Biosciences Clontech, San Jose, USA), was transfected into the hMSCs by electroporation using the Lonza 4D-Nucleofector™ system (Lonza, USA). hMSCs were transfected with 3 μg pIRES2-EGFP and pIRES2-mHCN2-EGFP vectors, and the efficiency of transfection was estimated by the number of green fluorescence-emitting cells (enhanced green fluorescence protein (EGFP) excitation 488 nm, emission 509 nm). The total number of green fluorescent cells 24–48 h after the incorporation of vectors revealed 40–50 % transfection efficiency. Transfected cells were cultured in hMSC growing medium (Poietics MSCGM, BioWhittaker, Lonza) at 37 °C in a 5 % CO_2_ humidified atmosphere. To select a pure population of mHCN2-expressing hMSCs, cells were exposed to 50 μM geneticin for an additional 5 days. Transfection efficiency was also confirmed by both quantitative real-time polymerase chain reaction (RT-qPCR) and enzyme-linked immunosorbent assay (ELISA).

### Investigation of mHCN2 expression by RT-qPCR

**Fig. 1:**
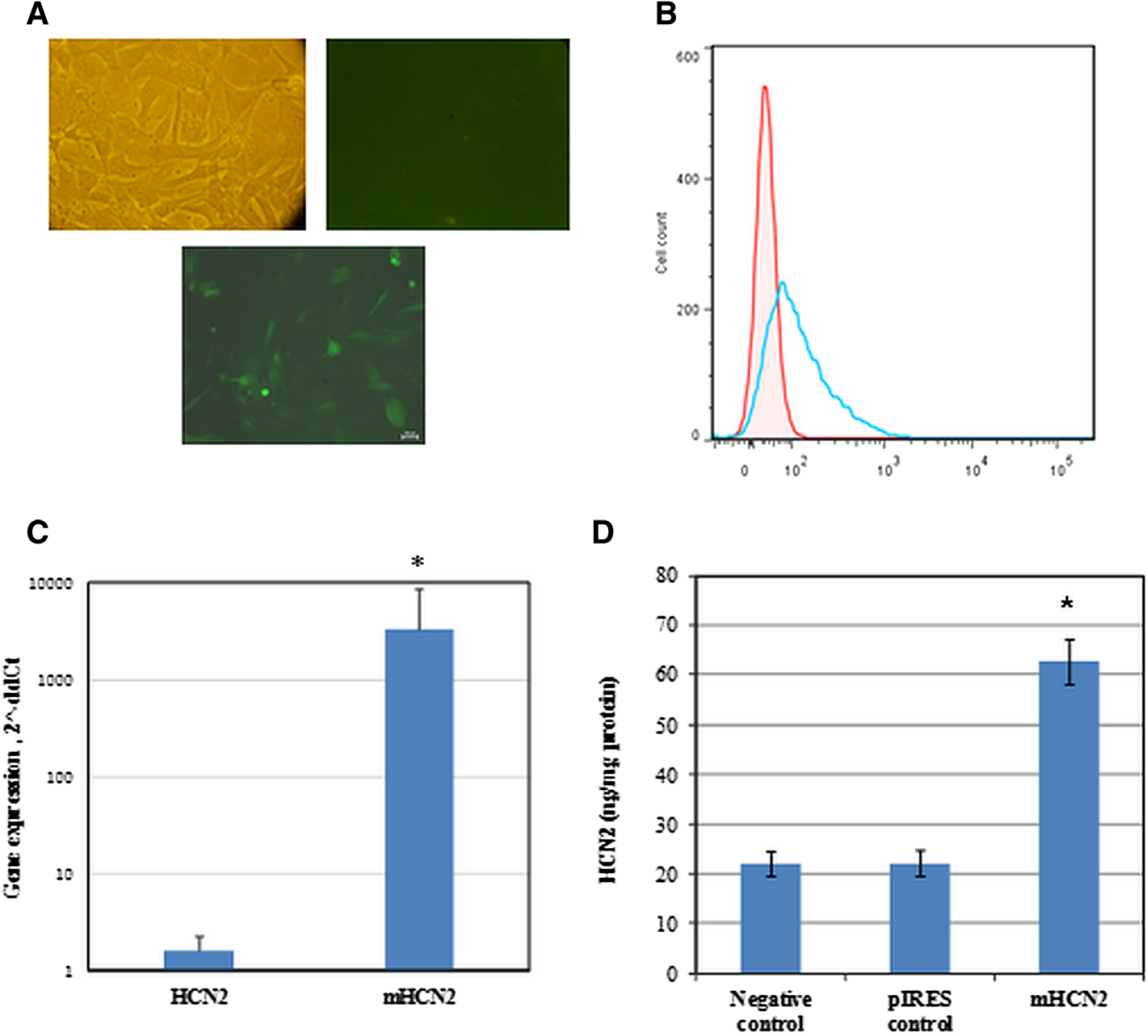
Efficiency of mouse HCN2 (*mHCN2*) transfection in hMSCs cells. **a** Primary evaluation of transfection efficiency by microscopy: *upper left panel*, light microscopy picture of hMSCs; *upper right panel,* fluorescent picture of not transfected cells; *lower panel*, fluorescent picture of mHCN2-transfected cells. Cells were investigated 48 h after the seeding. Magnification 20×. **b** Evaluation of transfection efficiency by flow cytometry. *Red*, fluorescence of cells before transfection; *blue*, fluorescence of cells after transfection. One representative graph is shown. **c** Expression of the mHCN2 gene in hMSCs was estimated by RT-qPCR. HCN2 column shows the level of endogenous human HCN2 in hMSCs cells estimated with cDNA against human HCN2. **d** Level of intracellular mHCN2 protein after transfection was estimated by ELISA and expressed as ng/mg protein. Protein was measured as described in the Methods section. Data are presented as means ± SD. The increased mHCN2 expression after transfection was significant at *p < 0.05

pIRES-EGFP- and pIRES-mHCN2-EGFP-transfected hMSCs were lysed, and their RNA was extracted and purified using RNeasy Mini Spin columns (Qiagen, USA) according to the manufacturer’s instructions. RNA concentration and purity were evaluated with a SpectraMAX i3 spectrophotometer (Molecular Devices, USA). RNA samples were treated with DNase I (Thermo Scientific™) and reversely transcribed with the Maxima®First Strand cDNA Synthesis Kit (Thermo Scientific™) according to the manufacturer’s protocols. PCRs were performed using Maxima® Probe qPCR Master Mix (2×) (Thermo Scientific™) and Stratagene MX-3005P detection instrument (Agilent Technologies, USA). The TaqMan® Gene Expression Assays (Applied Biosystems, USA) for *HCN2* (human, Hs00606903_m1), *mHCN2* (mouse, Mm00468538_m1), and *ACTB* (housekeeping gene, Hs01060665_g1) were used for gene expression analysis and separation of endogenous human HCN2 from transfected mHCN2. Expression of the mHCN2 gene after transfection was compared with the level of the endogenous human HCN2 gene in hMSCs (Fig. 1a). All reactions were run in triplicate starting with a denaturation step for 10 min at 95 °C followed by 40 cycles of 15 s at 95 °C for denaturation and 60 s for annealing and extension. The gene expression ratio (pIRES-mHCN2 vs. non-transfected cells) was calculated using the 2^-∆∆Ct^ equation. The efficiency of mHCN2 transfection was measured 5 days after the cell growth with 50 μM geneticin.

### Evaluation of mHCN2 protein expression by ELISA

Cells were lysed using three cycles of freezing-thawing. Before making measurements, all samples were kept on ice. mHCN2 expression after hMSC transfection was measured using an ELISA kit for the estimation of mouse potassium/sodium hyperpolarization-activated cyclic nucleotide-gated channel 2 (EIAab, cat. No.E15069m) following the manufacturer’s instructions. Absorbance was measured at 450 nm using a Spectramax plate reader. Three groups of cell lysates were investigated: pIRES-mHCN2-EGFP-expressing (positive control), pIRES-EGFP-transfected (control with transfection reagent), and non-transfected (negative control) hMSCs. The concentration of total protein in all tested groups was measured using the Bio-Rad DC Protein Kit according to the manufacturers’ instructions. Absorbance was read at 750 nm using a Spectramax plate reader. The final concentration of intracellular mHCN2 after transfection was expressed as ng/mg protein. The efficiency of mHCN2 protein expression in hMSCs was measured 5 days after cell growth with 50 μM geneticin.

### Patch-clamp and dye transfer measurements

For electrophysiological recordings, glass coverslips with hMSCs were transferred to the experimental chamber with constant flow-through perfusion, and mounted on the stage of an inverted microscope (Olympus IX81). Junctional conductance between hMSCs (abutted or connected through tunneling tubes (TT)) was measured using the dual whole-cell patch-clamp technique. Cells 1 and 2 of a cell pair were voltage clamped independently with the patch-clamp amplifier (MultiClamp 700B; Molecular Devices, Inc., USA) at the same holding potential (V_1_ = V_2_). Voltages and currents were digitized using the Digidata 1440A data acquisition system (Molecular Devices, Inc.) and acquired and analyzed using pClamp 10 software (Molecular Devices, Inc.). By stepping the voltage in cell 1 (ΔV_1_) and keeping the other constant, junctional current was measured as the change in current in the unstepped cell 2, I_j_ = ΔI_2_. Thus, g_j_ was obtained from the ratio –I_j_/ΔV_1_, where ΔV_1_ is equal to transjunctional voltage (V_j_), and a negative sign indicates that the junctional current measured in cell 2 is oppositely oriented to that measured in cell 1.

To examine whether cells residing on the opposite sides of Kapton® scaffold can couple through 3 μm diameter pores, non-transfected hMSCs were seeded on one side of the scaffold and 24 h later the mHCN2-transfected cells were seeded on the other side of the scaffold. After the attachment of transfected cells, DAPI dye (20 μM) was injected through the patch pipette into the mHCN2-transfected hMSC, and its transfer to the non-transfected cells residing on the other side of the scaffold was monitored by time lapse imaging at 37 °C in a humidified atmosphere of 5 % CO_2_ using an incubation system (INUBG2E-ONICS; Tokai Hit, Shizuoka-ken, Japan) with an incubator mounted on the stage of the microscope equipped with an Orca-R^2^ cooled digital camera (Hamamatsu Photonics K.K., Japan), fluorescence excitation system MT10 (Olympus Life Science Europa Gmbh, Hamburg, Germany), and XCELLENCE software (Olympus Soft Imaging Solutions Gmbh, München, Germany).

Patch pipettes were pulled from borosilicate glass capillary tubes with filaments. To minimize the effect of series resistance on the measurements of g_j_ [10], we maintained pipette resistances below 3 milli-ohms. Patch pipettes were pulled from borosilicate glass capillary tubes with filaments. Experiments were performed at room temperature in Krebs-Ringer solution (mM): NaCl, 140; KCl, 4; CaCl_2_, 2; MgCl_2_, 1; glucose, 5; pyruvate, 2; HEPES, 5 (pH = 7.4). Patch pipettes were filled with internal solution (mM): KCl, 130; Na aspartate, 10; MgATP, 3; MgCl_2_, 1; CaCl_2_, 0.2; EGTA, 2; HEPES, 5 (pH = 7.3). Patch-clamp measurements were performed 48–72 h after transfection.

### Cell viability-apoptosis assay

The number of viable cells, type of cell, and cell death, and stage of apoptosis of mHCN2-expressing hMSCs after selection with geneticin were analyzed using the Muse® Annexin V & dead cell assay (Merck Millipore) following the manufacturer’s instructions. This assay allows quantitative identification of live, early and late apoptotic, and dead cells by measuring the intensity of cell fluorescence. Briefly, cells transfected with the mHCN2 gene, only, with the pIRES vector, and non-transfected hMSCs were harvested with trypsin-EDTA and suspended in DMEM containing 10 % fetal calf serum. Cell suspension (100 μl) and Muse™ annexin V & dead cell reagent (100 μl; Annexin V and 7-AAD) were thoroughly mixed. Samples were incubated for 20 min in the dark. The Muse™ Cell Analyzer (Merc Millipore) was used to measure cell fluorescence. Cells were separated into groups according to the intensity of green (Annexin; early and late apoptotic cells) and red (7-AA; dead cells) fluorescence. Cell viability was tested 5 days after the transfected cell growth with 50 μM geneticin. Non-transfected cells were grown in parallel for the same time period.

### Measurements of cell proliferation and cell cycle

Proliferation measurements were performed using the CCK-8 kit (Dojindo Molecular Technologies, USA) according to the manufacturer's instructions. Briefly, after selection with geneticin, mHCN2- and pIRES-transfected hMSCs were grown for 5 days in 12-well plates. Similar numbers (25 × 10^3^) of non-transfected hMSCs were seeded in 12-well plates and allowed to attach for 24 h. Then, the number of proliferating cells in each cell group was measured every 24 h by adding a required volume of CCK-8 reagent with subsequent incubation for 3 h and measurement of absorption at 450 nm. Proliferation rates were measured for 72 h. Non-transfected hMSCs were grown and analyzed in parallel to the transfected ones.

Cell cycle was also measured 72 h after cell growth following the manufacturer’s instructions. Briefly, mHCN2- and pIRES-transfected, and non-transfected hMSCs were harvested with trypsin-EDTA. Cells were suspended in growth media containing 10 % fetal calf serum; 200 μl cells were added to each tube, centrifuged at 300 × g for 5 min and washed once with PBS. Then cells were suspended in 200 μl ice cold 70 % ethanol and incubated for 3 h at −20 °C. Cells were centrifuged at 300 × g for 5 min and washed once with PBS; 200 μl Muse™ Cell Cycle Reagent was added to each tube and incubated for 30 min at room temperature in the dark. The cell cycle reagent was a mixture of propidium iodide (PI) and RNAse A in respective proportions subsequently intercalating nuclear DNA. The assay allows identification and measurement of the percentage of cells in each cell cycle phase (G0/G1, S, and G2/M) according to the intensity of PI-based red fluorescence. The DNA Muse™ Cell Analyzer program was used to evaluate the results.

### Investigation of transcription factors by RT-qPCR

After selection with geneticin, pIRES-EGFP, pIRES-mHCN2-EGFP-transfected, and non-transfected hMSCs were lysed and RNA was extracted as described above. RNA was reversely transcribed with the RT^2^ First Strand Kit (Qiagen, USA) according to the manufacturer’s protocols. PCRs were performed using RT^2^ SYBR Green ROX qPCR Mastermix (Qiagen, USA) and the Stratagene MX-3005P detection instrument (Agilent Technologies). The Human Transcription Factors RT^2^ Profiler™ PCR Array (Qiagen, PAHS-075Z) was used to screen gene expression changes. Samples of three independent transfection experiments were investigated. Raw data were analyzed by the GeneGlobe Data Analysis Center platform (available online: http://www.qiagen.com/fo/shop/genes-and-pathways/data-analysis-center-overview-page/; Qiagen, USA). For the normalization of gene expression, the geometric mean of four threshold cycles (Ct) of reference genes was used. Gene expression ratio was calculated using the 2^-∆∆Ct^ equation. Data were statistically significant at *p* < 0.05. The expression of transcription factors in transfected cells was measured after cell growth with 50 μM geneticin for 5 days and compared with non-transfected hMSCs.

### Manufacture of Kapton® scaffold

Pores were micromachined in a commercially available 12.7-μm thickness polyimide Kapton® HN (DuPont, USA) film using FemtoLab workstation (Workshop of Photonics) and second harmonic (515 nm) of Yb:KGW femtosecond laser Pharos (Light Conversion). The diameters of the resulting pores were 1–3 μm, as determined by scanning electron microscope (Quanta 200 FEG) [11] under the low-vacuum mode.

### Immunocytochemistry

To detect the growth of mHCN2-transfected hMSCs and their possible transmigration through the Kapton® scaffold, 4 × 10^4^ non-transfected hMSC were labeled with PKH26 (red fluorescent cell linker kit; Sigma-Aldrich) following the manufacturer’s instructions and seeded on one side of a scaffold. The next day, 4 × 10^4^ mHCN2-transfected hMSCs (green fluorescence of EGFP) were seeded on the other side of the scaffold, which was then fixed on a specially designed cell crown holder. After 24 h of cell growth in media at 37 °C in a humidified atmosphere with 5 % CO_2_, cell were washed and fixed with 4 % paraformaldehyde and mounted in the Vectashield (Vector Labs, USA) containing DAPI for visualization of the nuclei. All samples were analyzed using a Leica TCS SP8 confocal microscope.

### Statistical analyses

All statistical analyses were performed using the SPSS package (version 19.0 for Windows; SPSS Inc., Chicago, IL, USA) and considered to be significant at the 5 % level. Differences between transfected and non-transfected cells were tested by analysis of variance (ANOVA) and Student’s *t* test. Data are presented as means ± SD.

### Ethical approval

All hMSC isolation procedures were approved by the Ethics Committee of Vilnius Regional Biomedical Research, Lithuania (No. 158200-14-741-257). All volunteers gave written consent and agreed with the investigational procedure of BM.

## Results

### Efficiency of hMSC transfection

**Fig. 2:**
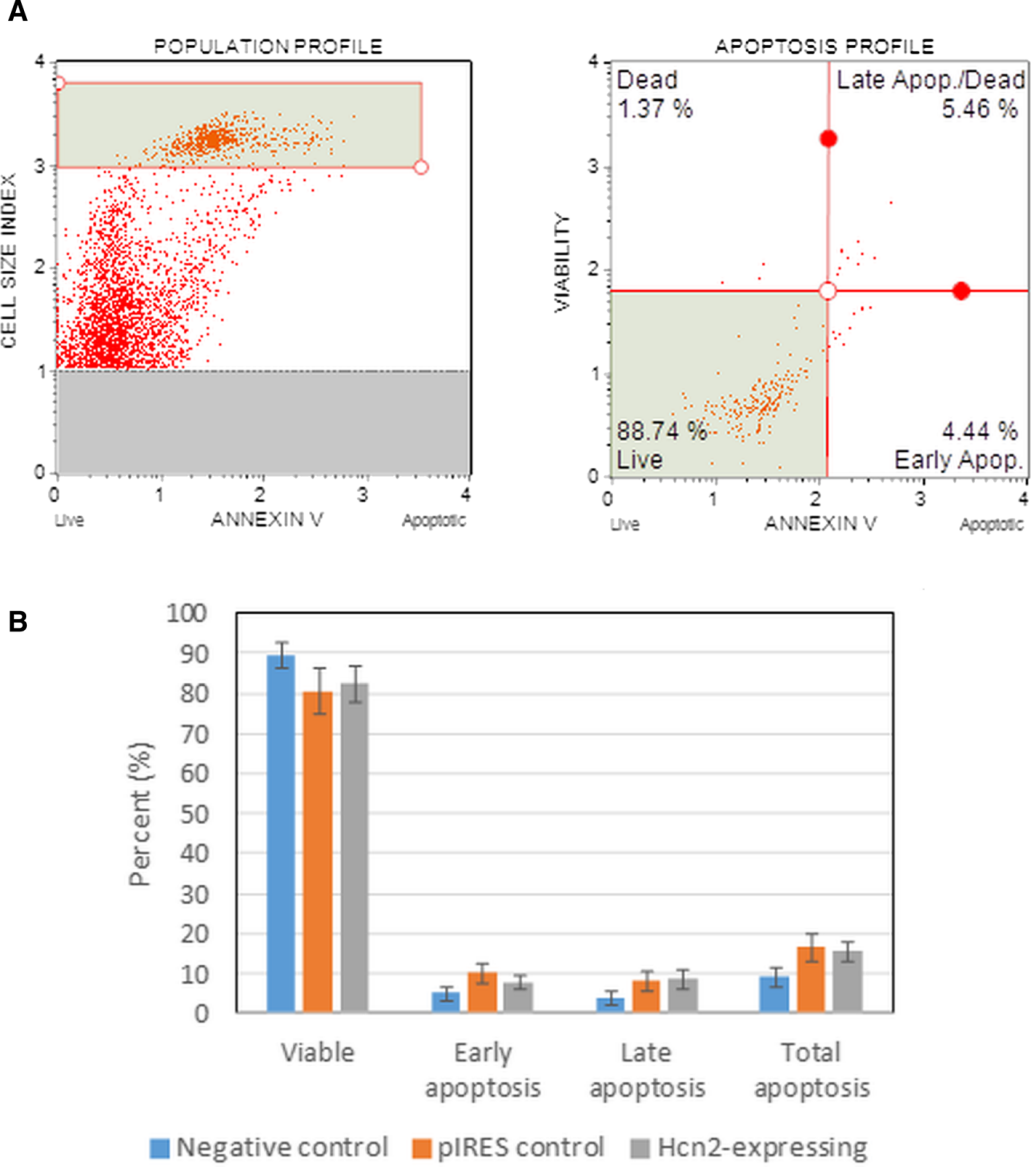
Measurement of apoptosis in transfected and non-transfected hMSCs. **a** Representative profiles of cell population (*left*) and apoptosis (*right*) of mHCN2-transfected cells. **b** Quantitative viability and apoptosis of non-transfected hMSCs (*negative control*), pIRES-EGFP (*pIRES control*) and pIRES-mHCN2-EGFP (mHCN2-transfected cells; *Hcn2-expressing*) transfected hMSCs. Data are presented as means ± SD from three independent transfection experiments. Transfected cells were investigated by Muse® equipment 5 days after their growth with 50 μM geneticin. Non-transfected cells grew and were investigated in parallel. The induction of apoptosis in transfected compared to non-transfected cells was statistically insignificant

The efficiency of transfection with pIRES2-EGFP and pIRES2-HCN2-EGFP vectors was evaluated by fluorescence microscopy, RT-PCR, and ELISA. Data in Fig. 1a show that non-transfected cells did not have green fluorescence, whereas transfected cells had green fluorescence. The efficiency of plasmid incorporation has also been confirmed by flow cytometry (Fig. 1b). mHCN2 gene expression was also confirmed by RT-PCR (Fig. 1c). mHCN2 protein expression was investigated by ELISA and expressed as ng/mg protein (Fig. 1d). Representative profiles of cell population and apoptosis of mHCN2-transfected cells analyzed by Muse™ Annexin V and Dead cell reagent is demonstrated in Fig. 2a. The percentage of early, late, and total apoptosis in cells from three independent transfection experiments is shown in Fig. 2b. Induction of necrosis after transfection was negligible. Cells transfected with the pIRES2-EGFP and with the pIRES2-mHCN2-EGFP vectors showed similar levels of live, early, and late apoptosis, which suggests that cell apoptosis was induced by the electroporation procedure, which could damage cell membranes, rather than by the mHCN2 gene itself.

### I_f_ and cell to cell coupling

**Fig. 3:**
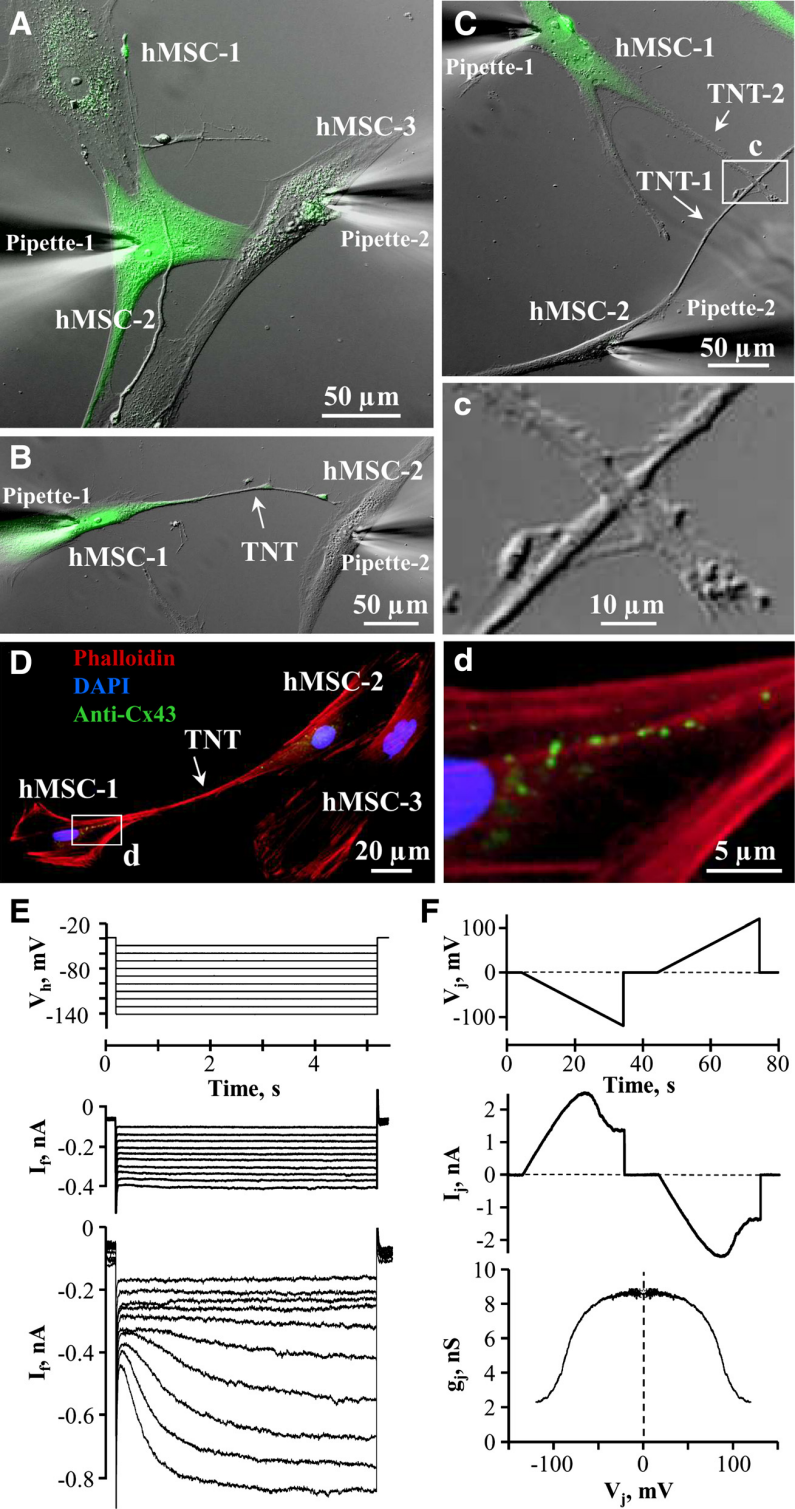
Current generation by mHCN2-expressing cells and their electrical coupling. Human mesenchymal stem cells (*hMSCs*) expressing mHCN2 (*green*) abutted (**a**) and connected through tunneling nanotubes (*TNTs*) (**b**, **c**). (inset c) GJ-based electrical coupling between cells also could be achieved by intersection of lamellipodia extensions. **d** and inset **d** TNTs between hMSCs containing F-actin, and Cx43-based GJs between hMSC-1 and the lamellipodium of hMSC-2. *I*_f_ current was measured in mHCN2-expressing hMSCs (**e**, *lower panel*) by hyperpolarizing the cells from V_h_ = −40 mV for 5 s to voltages between −50 and −140 mV in 10 mV increments (**e**, *upper panel*). *I*_f_ current was absent in non-transfected or transfected with empty vector hMSCs (**e**, *middle panel*). **f** Typical experiment showing the measurement of electrical coupling between two hMSCs connected through TNT (shown in **b**). g_T_/V_T_ plot (**f**, *lower panel*) was obtained by measuring the I_j_ response (**f**, *middle panel*) in the hMSC-2 to the voltage ramp of negative polarity from 0 to −120 mV (**f**, *upper panel*) applied to the hMSC-1 with its symmetric counterpart at positive V_j_s. The co-culture of mHCN2-transfected (green fluorescence) and non-transfected hMSCs (not fluorescent) was investigated

The expression of functional mHCN2 channels and electrical coupling between abutted hMSCs (Fig. 3A) or hMSCs connected through tunneling nanotubes (TNTs) (Figs. 3B and C) were examined by dual whole-cell patch-clamp measurements. The co-culture of mHCN2-transfected (green) and control hMSCs (not fluorescent) was investigated. TNTs formed by the lamellipodium outgrowth mechanism formed the gap junction (GJ)-based electrical coupling between the hMSCs that also could be achieved by intersection of lamellipodia extensions (inset c). TNTs between hMSCs contained F-actin and Cx43 GJs at the interface of hMSC-1 and the lamellipodium ending of hMSC-2 (Fig. 3D and inset d).

Non-transfected hMSCs did not exhibit time-dependent hyperpolarization (Fig. 3E, upper panel) activated current I_f_ (Fig. 3E, middle panel), whereas mHCN2 expressing hMSCs showed I_f_ current (Fig. 3E, lower panel). In the mHCN2-transfected hMSCs, I_f_ was nearly fully activated at −140 mV (504 ± 130 pA; *n* = 4) with an activation threshold of −90 mV. Fig. 3F (lower panel) displays the g_j_/V_j_ plot obtained by measuring the I_j_ response (Fig. 3F, middle panel) in the hMSC-2 to the voltage ramp (Fig. 3F, upper panel) applied to the hMSC-1 with its symmetric counterpart at positive V_j_s. The presence of voltage gating indicates that the cells established electrical coupling by forming functional GJs. The measured g_j_ between six abutted cell pairs was 37.14 ± 3.61 nS and g_T_ between five cell pairs connected through TNTs was 1.15 ± 0.25 nS.

### Cell cycle of mHCN2-expressing hMSCs

**Fig. 4:**
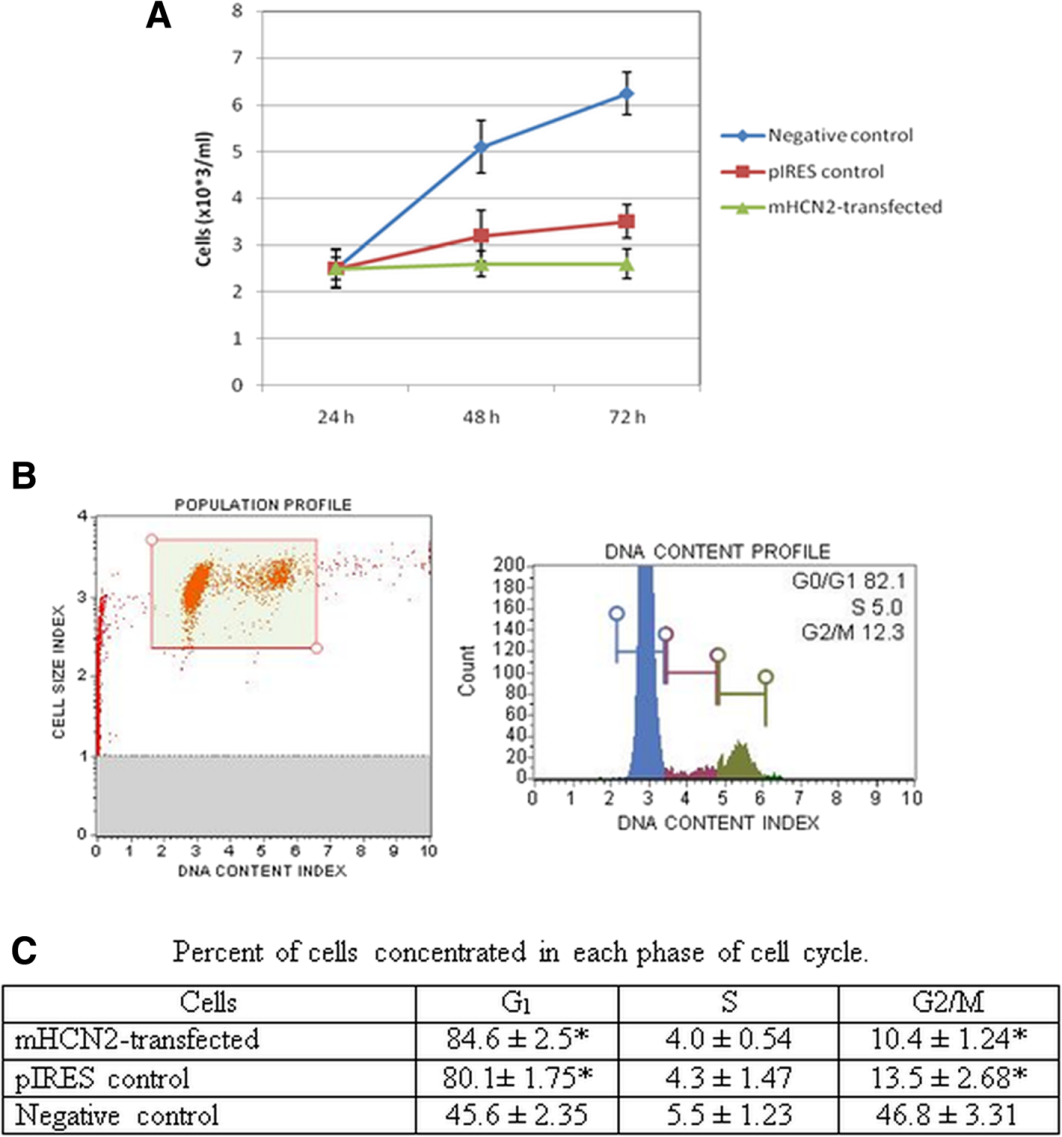
Proliferation and cell cycle of transfected and non-transfected hMSCs. **a** Proliferation rate of non-transfected (*negative control*), pIRES-EGFP-transfected (*pIRES control*) and pIRES-mHCN2-EGFP-transfected (*mHCN2-transfected*) hMSCs over 72 h. Control, pIRES-, and mHCN2-transfected cells were investigated after their growth with geneticin for 5 days. Changes in proliferation rate were significant (*p* < 0.05) compared to the non-transfected cells. **b** Representative profiles of cell population (*left*) and DNA content (*right*) of mHCN2-transfected hMSCs. **c** Summarized quantitative percentage of each cell cycle phase. Data are presented as means ± SD. *Data were significant compared to the negative control at *p* < 0.05. Proliferation of transfected cells was measured 5 days after their growth with geneticin (50 μM). Non-transfected cells grew for 72 h and were analyzed in parallel. Data are presented as means ± SD

Both types of transfected cells (with empty pIRES vector and with mHCN2 gene) showed significantly downregulated proliferation rates compared to the non-transfected hMSCs (Fig. 4a). Moreover, the mHCN2-transfected cells showed significantly lower proliferation rates compared to the pIRES-transfected cells (Fig. 4a). This finding led us to analyze the cell cycle and possible mechanisms involved in its regulation. Data in Fig. 4b show representative population and DNA content of one mHCN-2 transfection experiment, whereas data in Fig. 4c demonstrate summarized data from three independent transfection experiments compared to the non-transfected cells. mHCN2- and pIRES-transfected hMSCs were significantly concentrated in the G1 phase with low numbers of cells in G2/M phase, whereas non-transfected hMSCs had an almost equal distribution between G1 and G2/M phases (Fig. 4c). The mHCN2-expressing cells showed slightly stronger downregulation of the cell cycle compared to the pIRES-transfected cells. Cell cycle of transfected cells was measured after selection by 50 μM geneticin.

### Transcription factors regulating cell cycle of mHCN2-expressing hMSCs

**Fig. 5:**
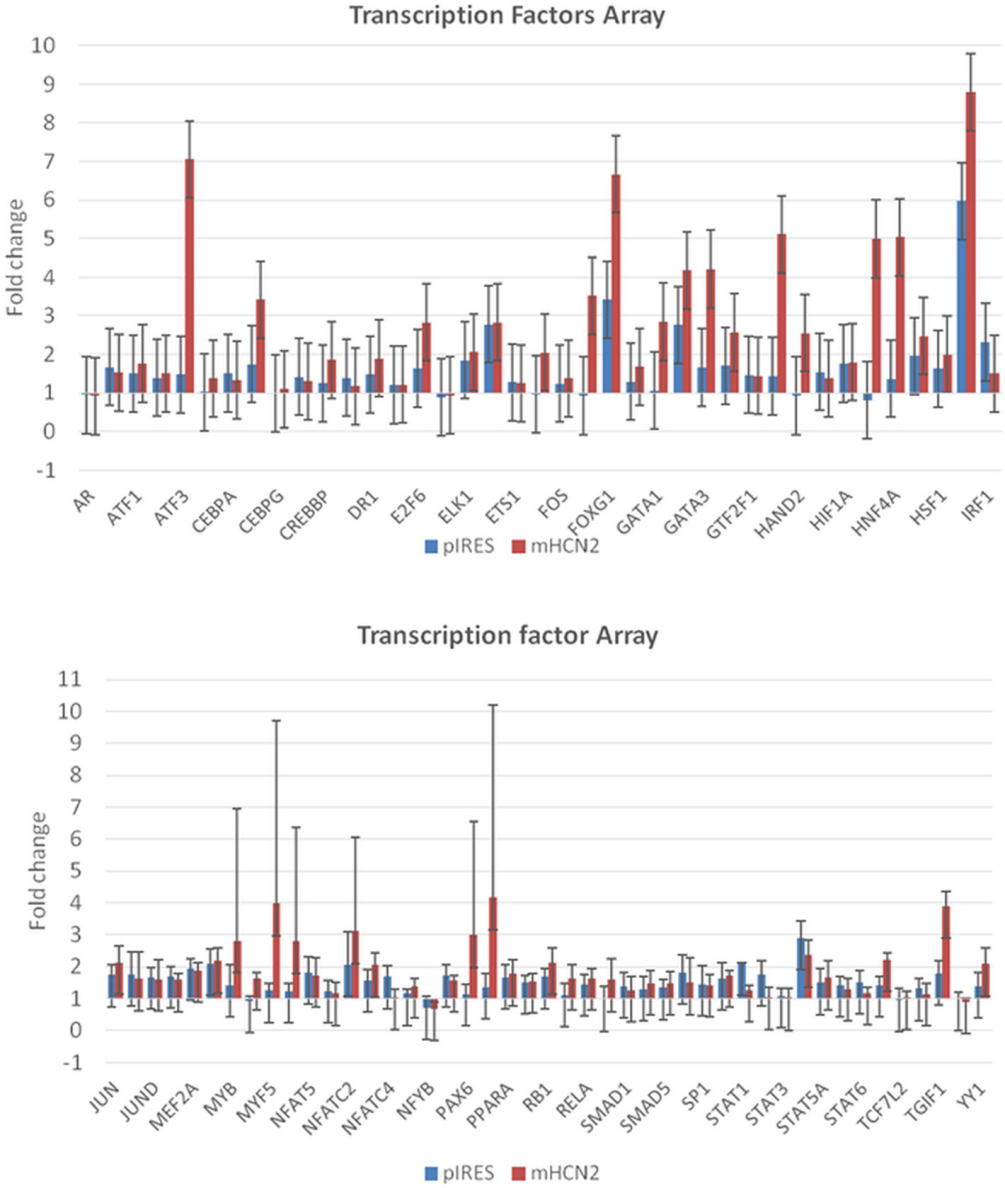
Panels of changed activities of transcription factor arrays after transfection. Folds of change in activities of transcription factors after pIRES- and mHCN2-transfections were compared to non-transfected cells. Data are presented as mean values of fold-changes obtained during three independent experiments. The statistically significant activation of nine transcription factors after mHCN2 transfection compared to pIRES transfection is presented in Table 1

Data from the cell cycle experiments inspired us to investigate further mechanisms regulating the cell cycle after transfection. For this purpose, we investigated an array of transcription factors and their changes after both types of transfection (pIRES and mHCN2) compared with non-transfected hMSCs. Data presented in Fig. 5 show changes in expression of transcription factors measured in pIRES- and mHCN2-transfected cells compared to the negative control (non-transfected hMSCs). Further statistical analysis of PCR data revealed that mHCN2-transfected cells significantly upregulated nine transcription factors involved in cell cycle regulation compared to the pIRES-transfected cells (Table 1). It also demonstrates that the activation of the nine transcription factors is a result of the mHCN2 gene and not the transfection procedure. The most significantly upregulated factor in mHCN2-transfected cells was activating transcription factor ATF3, a member of the cAMP response element-binding (CREB) protein family of transcription factors (7.11-fold, *p* = 0.00056). Other cell cycle regulating transcription factors were upregulated from 1.6- to 2.97-fold (*p* < 0.05; Table 1).

**Table 1:** Statistically evaluated impact of mouse HCN2 to the expression of transcription factors

No	Gene	Main impact to cell cycle regulation	Fold-change	*P* value
1.	ATF3	Delays G1 to S transition [29]	7.11	0.00056
2.	ETS2	Promotes G2/M phase [46]	2.79	0.02
3.	GTF2B	Regulates G1 phase [33]	1.9	0.01
4.	ID1	Promotes G1/S phase [37]	1.86	0.044
5.	MYC	Promotes G1/S phase [36]	2.3	0.0028
6.	NFATC3	Promotes G1/S phase [41]	1.6	0.02
7.	REL	Promotes G0 toG1 transition [45]	1.94	0.014
8.	TBP	Delays G2/M phase [24]	2.04	0.012
9.	TGIF1	Promotes G0 toG1 transition [22]	2.97	0.025

### Growth of mHCN2-expressing cells on porous scaffold

**Fig. 6:**
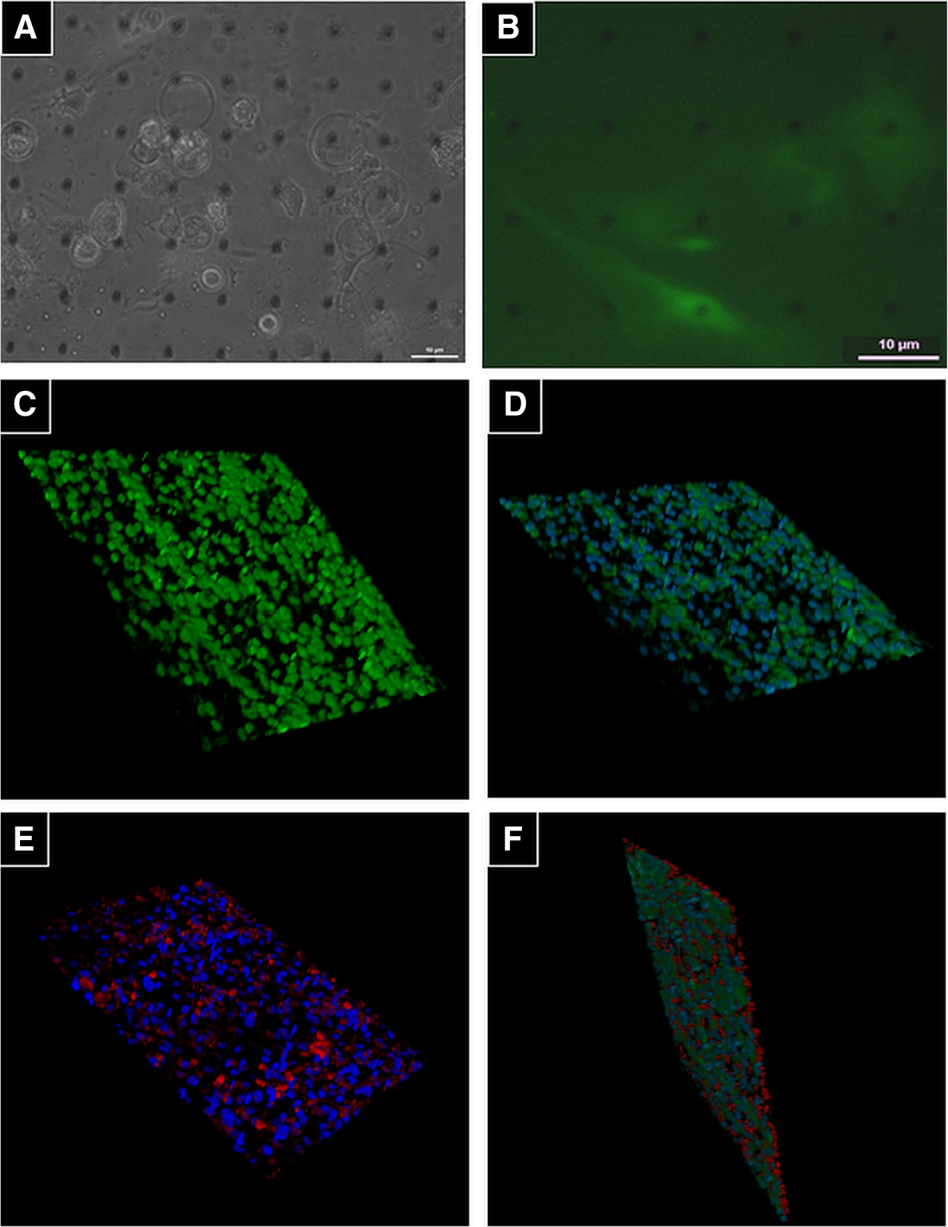
Growth of mHCN2-expressing cells on Kapton® scaffold. **a** Light microscopy image of mHCN2 cells grown on the Kapton® scaffold. Magnification 20×. **b** Fluorescent image of mHCN2-transfected hMSC grown on the Kapton® scaffold. Magnification 20×. **c** Confocal three-dimensional image of mHCN2-transfected cells (*green*). Magnification 20×. *Scale bar* 10 μm. **d** Confocal three-dimensional image of mHCN2-transfected cells (*green*) and nucleus (*blue*). Magnification 20×. *Scale bar* 10 μm. **e** Confocal three-dimensional image of non-transfected hMSCs stained with PKH26 (*red*) and nucleus (*blue*). Magnification 20×. **f** Confocal three-dimensional image of mHCN2-transfected cells (*green*) on the one side of Kapton® scaffold and hMSC stained with PKH26 (*red*) on the opposite side. Nuclei were stained *blue*. Magnification 20×

**Fig. 7:**
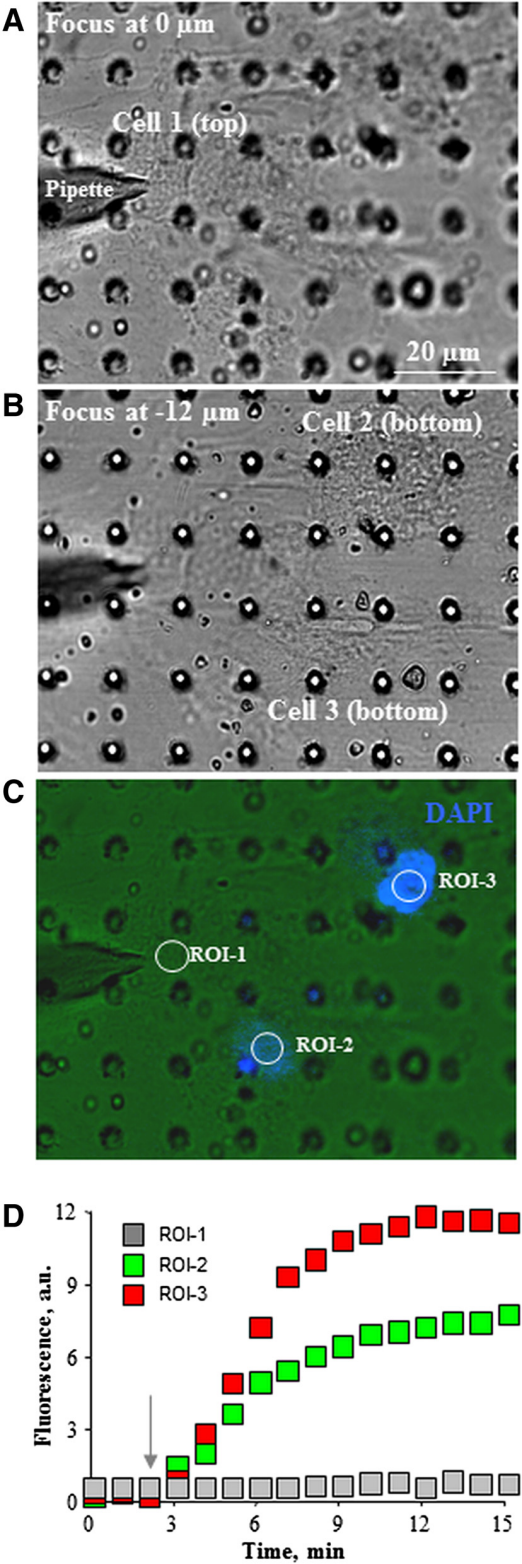
Intercellular coupling of cells growing on the opposite sides of Kapton® scaffold. **a** DAPI (20 μM) was injected through the patch pipette into the mHCN2-transfected cell 1 (nucleus encircled in **c**) residing on the top side of Kapton® scaffold. **b** Non-transfected hMSCs (cell 2 and cell 3) residing on the bottom side of the scaffold (nucleus encircled in **c**). **c** Transfer of DAPI dye from the donor cell 1 to the recipient cell 2 and cell 3. Note that the fluorescence of DAPI-stained nucleus in cell 1 could not be demonstrated because of the impermeability of Kapton® scaffold to UV light. **d** DAPI accumulation kinetics measured in the regions of interest (*ROI*) situated on the nuclei of respective cells. *Arrow* indicates the onset of dye application to cell 1

Stem cells directly grafted into heart tissue or injected through the vessels may be released due to the strong contraction of heart muscle or as a response to signaling molecules. Therefore, we investigated the possibility of enclosing cells in a cage/scaffold with particular pore sizes that could be impermeable for cells but allow passing of nutrients and/or anchorage-dependent cell to cell communication. Data from other authors showed that the best pore size for cell growth without transmigration through the scaffold could be up to 3 μm in diameter [12]. With this in mind, we investigated 8.9–16.5 μm thick Kapton® scaffold with 1–3 μm pores of conical shape. Data in Fig. 6 show that mHCN2-expressing cells were able to attach and grow on the Kapton® scaffold without transmigration. Confocal micrographs demonstrate that the same mHCN2-transfected cells were able to grow on one side (Fig. 6a-d) and non-transfected hMSCs stained with PKH26 on the other side of scaffold (Fig. 6e). Both types of the above-mentioned cells could grow on different sides of the scaffold without transmigration (Fig. 6f). Moreover, the mHCN2-expressing hMSCs growing on the one side of the Kapton® scaffold (cell 1 in Fig. 7a; nucleus encircled in Fig. 7c) can establish the intercellular communication with the non-transfected hMSCs growing on the other side of the scaffold (cells 2 and 3 in Fig. 7b; nuclei encircled in Fig. 7c). DAPI dye injected through the patch pipette into cell 1 was transferred to cells 2 and 3, confirming that cells residing on the opposite sides of the scaffold can couple to each other through 3-μm pores. Note that nucleus staining in the DAPI-injected cell 1 is not visible because the Kapton® scaffold is impermeable to the UV light used for excitation of DAPI fluorescence, while nuclei staining in the recipient cell 2 and cell 3 are obvious, and DAPI accumulation kinetics in these cells is shown in Fig. 7d.

## Discussion

The major requirements for generating functional biological pacemakers are construction of viable, properly functioning and proliferating cells capable of generating the pacemaker current and growing on the porous scaffolds. In this study, we have demonstrated that mHCN2-transfected hMSCs expressed the mHCN2 channel protein and exhibited I_f_ current necessary for cardiac stimulation. However, the capacitances of transfected hMSCs varied from 100 to 200 pF, and I_f_ densities in our experiments were limited to several pA/pF. This was presumably due to insufficient translation and/or not completed insertion of mHCN2 channels into the cell membrane after transfection. The mHCN2-transfected MSCs preserved their viability, generated pacemaker current, grew on the porous Kapton® scaffolds with 1–3 μm diameter pore arrays, and established intercellular communication between opposite sides of the scaffold without transmigration through the pores. The polyimide films demonstrated suitable mechanical and thermal properties, and good biocompatibility, and have already been successfully applied to a vast range of biomedical investigations [13–17]. In parallel to the formation of functionally active HCN channels, the mHCN2-transfected MSCs expressed Cx43 necessary for communication through Cx43-based GJ channels and F-actin containing TNTs necessary for the biological pacemaker functioning. The collaboration of functional GJs and HCN2 channels as a pacemaker unit in heterologous cell pairs has been shown elsewhere [18].

Importantly, data from this study show, for the first time, that the proliferation rate of mHCN2-transfected cells was significantly downregulated, mainly by the nine transcription factors that controlled the cell cycle. Our findings show mHCN2-expressing hMSCs have low proliferative activity that could be vitally important for the proper and long-term function of heart-stimulating cells. Other authors also revealed that quiescence and self renewal are critical for stem cell pool preservation and long-term engraftment potential [19]. Highly proliferating cells do not have enough time for cell renewal, and eventually their supply might become exhausted. On the other hand, given the extracellular stimuli and fulfillment of various regenerating functions, stem cells cannot be quiescent all the time [20]. Data from this study show that the balance between cell cycle and functional activity of mHCN2-transfected hMSCs is important for the biological pacemaker. The ability to modify the balance between the activation of cell cycle-regulating transcription factors and HCN channel proteins could be a useful tool in the field of biological pacing.

TG-interacting factor 1 (TGIF1) is a transcriptional repressor and necessary factor modulating the balance between cell quiescence, self renewal, and differentiation. TGIF1 knockdown in myeloid progenitors affected cell proliferation and induced cell cycle blocking at the G0 stage [21]. Another study also showed that TGIF1 knockout resulted in increased quiescence of hematopoietic stem cells, their self-renewal, and a tendency to reside in the G0 state [22]. On the other hand, the transcription factor c-Rel protein, a member of the NFkB transcription factor family, stimulates the cell cycle as well as the various other intracellular functions. Binding of NFkB to the cell DNA is rapidly induced by serum growth factors and stimulates the G0 to G1 transition in mouse fibroblasts [23]. Additionally, it was shown that *c-rel* is also induced by serum in quiescent fibroblasts and the level of *c-rel* transcripts decreases to nearly the basal level 3 h after stimulation [24]. This finding suggests that *c-rel* is involved in the transition from the G0 to G1 phase. C-Rel was also found in the S phase, whereas its level decreased when cells entered the G2 phase [25]. Our data show that increased expression of TGIF1 and c-Rel by 2.97- and 1.94-fold, respectively, can be responsible for the advancement of mHCN2-expressing cells from the G0 stage to further phases of the cell cycle.

The next step in cell cycle regulation is controlled transition of mHCN2-transfected cells into the G1 phase. This step is strongly regulated by ATF3, which belongs to the family of CREB transcription factors and is a stress-inducible gene [26]. ATF3 has an anti-apoptotic effect and inhibits adriamycin-induced apoptosis in primary cardiomyocytes [27]. Furthermore, chick embryo fibroblasts stably expressing ATF3 grew better under low serum conditions [28]. It was also shown that ATF3+/+ fibroblasts more slowly transitioned from the G1 to S phase, suggesting a growth cycle control in the G1 phase [29, 30]. Data from this study show that significant activation of ATF3 (7.11-fold, *p* < 0.001) after mHCN2 transfection in hMSCs can control the cell cycle in the G1 phase and support better cell viability. The strong activation of ATF3 might also show the formation of functionally active mHCN2 channels which are cAMP sensitive. Another factor important for the control of the G1 phase is a general transcription factor IIB (GTF2B), which stimulates transcription through the stabilization of RNA polymerase II, initiating the DNA-TBA (TATA-binding) complexes [31]. It was shown that TF2B can also act as an autoacetyltransferase, which is important for TFIIB acetylation, stabilization, and activation of cell transcription [32]. GTF2B activation is mainly observed in the G1 phase, not in the M phase, suggesting suppression of cell proliferation [33]. Our data show that when GTF2B in mHCN2-transfected hMSCs was activated 1.9-fold, G1 to S transition was delayed similarly to ATF3.

The helix-loop-helix protein ID1 is important for cell “stemness” and is scarcely expressed in normal adult differentiated tissues, whereas it is abundant in proliferating tissues [34]. Under low serum conditions, ectopic expression of ID1, but not ID2, supported proliferation of mammary epithelial cells [35]. Downregulation of ID1, similar to c-Myc, decreased expression of cyclins D1 and E. The same authors suggested that ID1 is downstream of c-Myc in regulation of cell proliferation. Consistent with this idea, both c-Myc and ID1 are necessary for sufficient G1 to S progression [36, 37]. Another transcription factor, the nuclear factor of activated T cells (NFAT), belongs to a family of transcription factors that has been foremost identified in immune cells and later on in a wide range of cell types and tissues [38, 39]. NFAT is constitutively expressed in resting cells, whereas the activation of its receptor is related to the mobilization of calcium and subsequent cell activation [40]. It was also shown that calcium signals stimulate progression of the cell cycle and promote transition of the G1/S phase [41]. Our data show that ID1, c-Myc, and NFATC3 were activated 1.86-, 2.3- and 1.6-fold (*p* < 0.05), respectively, and could be involved in transition of the G1/S phase.

The last group of transcription factors controls the G2/M phases. The TATA-binding protein (TBP) is a universal transcription factor required for the eukaryotic RNA polymerase. TBP like-null chicken cells exhibited 20 % elevated cell cycle progression due to shortening of the G2 phase [42], and TBP induces a delay in the G2/M transition which subsequently delays cell proliferation [43]. Moreover, TBP in stressed cells instead of transcription regulation preferentially binds and repairs injured DNA [44]. This study shows that 2.04-fold activation of TBP after mHCN2 transfection may delay cell entrance to the G2-M phase. On the other hand, the member of the E26 transformation-specific (ETS) family member ETS2, the winged helix-turn-helix DNA-binding domain, has been found to be a regulator of cdc2 expression necessary for the G2/M phase and better cell growth under stress conditions [45]. Moreover, ETS2 activation is necessary for trophoblast stem cell self-renewal and is a vitally important factor for the survival of mouse embryos [46]. Data from this study demonstrate that 2.79-fold ETS2 activation will slightly stimulate mHCN2-expressing cells to proliferate, and might be important for better cell survival.

## Conclusions

The results of this study show that mHCN2-transfected hMSCs preserved high cell viability and functional activity necessary for cardiac stimulation: mHCN2-expressing cells had low proliferative activity due to the downregulation of the cell cycle and cell concentration in the G1 phase (~85 %); and generated I_f_ current and made anchorage-dependent connection with other cells without transmigration through a 12.7-μm thick Kapton® HN film with micromachined 1–3 μm diameter pores. Insertion of mHCN2 gene into hMSCs activates nine transcription factors that control each phase of the cell cycle, subsequently downregulating cell proliferation. The strongest activation of cAMP-responsive transcription factor ATF3 suggests its particular role in the G1 phase arrest. Additionally, a strong activation of ATF3 in mHCN2-transfected cells could be a marker of formation of functionally active HCN channels which are also cAMP-dependent. This study shows that mHCN2-transfected BM-derived hMSCs are appropriate for the further generation of functional biopacemakers.

## Abbreviations

AMP: adenosine monophosphate
ATF3: activating transcription factor 3
BM: bone marrow
cdc2: cell division cycle protein 2 homolog
c-Myc: v-myc myelocytomatosis viral oncogene homolog (avian)
CREB: cAMP response element binding protein
c-Rel: NF-kappa-B heterodimer RELA/p65.
Cx43: connexin 43
DAPI: 4′,6-diamidino-2-phenylindole
DMEM: Dulbecco’s modified Eagle’s medium
EGFP: enhanced green fluorescent protein
ELISA: enzyme-linked immunosorbent assay
ESC: embryonic stem cell
ETS: E26 transformation-specific
GJ: gap junction
g_j_: gap junction conductance
GTF2B: general transcription factor IIB
HCN2: potassium/sodium hyperpolarization-activated cyclic nucleotide-gated ion channel 2
hMSC: human mesenchymal stem cell
ID1 and ID2: DNA-binding protein inhibitors
I_f_: funny current
mHCN2: mouse HCN2
MSC: mesenchymal stem cell
NFAT: nuclear factor of activated T cells
NFkB: nuclear factor-κB
PBS: phosphate-buffered saline
PI: propidium iodide
ROI: region of interest
RT-qPCR: quantitative real-time polymerase chain reaction
TBP: TATA-binding protein
TGIF1: TG-interacting factor 1
TNT: tunneling nanotube
TT: tunneling tubes
